# *OsATG8c*-Mediated Increased Autophagy Regulates the Yield and Nitrogen Use Efficiency in Rice

**DOI:** 10.3390/ijms20194956

**Published:** 2019-10-08

**Authors:** Xiaoxi Zhen, Xin Li, Jinlei Yu, Fan Xu

**Affiliations:** Key Laboratory of Northern Japonica Rice Genetics and Breeding, Ministry of Education and Liaoning Province, Key Laboratory of Northeast Rice Biology and Genetics and Breeding, Ministry of Agriculture, Rice Research Institute of Shenyang Agricultural University, Shenyang 110866, China; xiaoxizhen1991@163.com (X.Z.);

**Keywords:** *OsATG8c*, nitrogen use efficiency (NUE), yield, autophagy, rice

## Abstract

Autophagy, a conserved pathway in eukaryotes, degrades and recycles cellular components, thus playing an important role in nitrogen (N) remobilization. N plays an important role in the growth and development of plants, which also affects plant yield and quality. In this research, it was found that the transcriptional level of a core autophagy gene of rice (*Oryza sativa*), *OsATG8c*, was increased during N starvation conditions. It was found that the overexpression of *OsATG8c* significantly enhanced the activity of autophagy and that the number of autophagosomes, dwarfed the plant height and increased the effective tillers’ number and yield. The nitrogen uptake efficiency (NUpE) and nitrogen use efficiency (NUE) significantly increased in the transgenic rice under both optimal and suboptimal N conditions. Based on our results, *OsATG8c* is considered to be a good candidate gene for increasing NUE, especially under suboptimal field conditions.

## 1. Introduction

Autophagy is a conserved vacuolar degradation pathway by which cells recycle components, including unwanted macromolecular substances or damaged organelles. The nutrients are mobilized and reused for the maintenance of cellular processes and adaptation to stress [1]. The first AuTophaGy-related gene (*ATG* gene) was identified in yeast [2]. Subsequently, many *ATG* genes have also been characterized in different species, including mammals and plants [3,4,5]. In Arabidopsis (*Arabidopsis thaliana*), most *ATG* genes are transcriptionally up-regulated during leaf senescence and nutrient starvation [6,7,8]. Among mammals, yeasts and plants, the identified proteins are responsible for the core autophagic mechanism [9].

Autophagy controls the remobilization of nitrogen (N) from leaves to seeds and was first discovered in Arabidopsis *atg* mutants [10]. The Arabidopsis wild type (WT) and *atg* mutants (*atg18a* RNAi, *atg5*, and *atg9*) were fed ^15^NO_3_^−^ during the vegetative growth stage. Under N stress, all *atg* mutants showed that ^15^N remobilization sharply decreased compared to WT, and the nitrogen use efficiency (NUE) from leaves to seeds decreased significantly, by nearly to 50% [11]. In addition, some studies revealed that the *atg* mutants (Arabidopsis, maize, and rice) have a premature senescence phenotype, which changes the metabolome and protein accumulation of cells, reducing the biomass, yield, and tolerance to biotic and abiotic stress [12,13,14]. Through a ^15^N pulse-chase analysis it was shown, that the maize *atg* mutant is unable to mobilize N from senescent leaves to seeds and has a reduced NUE compared with control plants [15]. Subsequent studies have also found that autophagy takes part and plays an important role in the formation of eukaryotic proteins and membranes [16].

Plants are sedentary and cannot move to acquire nutrients and minerals as needed. Their survival depends on their ability to consume the mineral nutrients available in the rhizosphere and metabolize, recycle, and conserve them efficiently during their lifespan [17,18]. Nitrogen (N) is the most important nutrient for plant development and growth and is also a vital component of macromolecules in cells. In cereal crops, N mobilization from aging leaves has an important effect on the grain yield and quality [19,20,21]. The grain yield of rice is mainly determined by the number of panicles per plant, the number of grains per panicle, and the grain weight, while the panicle number is dependent on the rice tillering ability [22]. Tillering in crops is regulated by genetic, hormonal, developmental, and environmental factors [23,24]. Previous studies have identified numerous genes related to tillering regulation in rice. *MOC1* was the first gene characterized for rice tillering, and it functions to initiate axillary buds [25]. *OsTB1/FC1* and *OsSPL14* have been reported to repress rice tillering [26,27]. Genes related to the biosynthesis and signaling of strigolactones, such as the DWARF genes *D3* [28], *D17/HTD1* [29], *D10/OsMAX4* [30], *D14*/*HTD2* [31], *D27* [32], and *D53* [33] have been suggested to be involved in tillering regulation in rice. The cytokinin oxidase/dehydrogenase (CKX) enzyme coding gene *OsCKX2* negatively regulates the tiller number and grain yield in rice [34,35], whereas the indole-3-acetic acid (IAA)-glucose synthase gene *OsIAGLU* positively affects tillering [36]. Recent research shows that the overexpression of *OsSta2* and the rice nitrate-transporter gene *OsNPF7.2* can increase the tiller number and yield in rice [37,38], while reduced expression of *OsAAP3* can also significantly increase the tiller number [39].

ATG8 is a ubiquitin-like protein, which is located on the membrane of the autophagosome [40,41]. ATG8 is often used as a reliable marker of autophagic activity in plants and animals [42,43]. Due to providing a docking site for autophagic receptors, which contains the ATG8 interaction motif (AIM) and selects the degraded cargo, ATG8 acts as a central component in autophagy. The first identified *ATG8* in rice was *OsATG8a*, which interacts with *ATG4* [44]. *OsATG8a*, *OsATG8b*, and *OsATG8c* have high homology, while *OsATG8d* is similar to *AtATG8i* [45,46]. It was shown that the overexpression of *ATG8*s from different plants, such as *AtAtg8f*, *MdATG8i*, *GmATG8c*, and *SiATG8a*, led to better growth and promoted tolerance to N-limited stress [47,48,49,50,51]. Recently, it was showed that the *AtATG8*s (*AtATG8a*, *AtATG8e*, *AtATG8f*, or *AtATG8g*)-overexpressing transgenic Arabidopsis increased the autophagosome number, stimulated autophagic activity, and increased the N remobilization efficiency (NRE) under full N conditions but did not affect the yield and biomass [52]. Our own contemporaneous research also found that the overexpression of *OsATG8a* in rice could increase the autophagic flux, grain yield, and NUE [53]. 

In this study, we investigated the potential functions of the *OsATG8c* gene (LOC_*Os08g09240*) of rice. This gene has a 363 bp coding sequence (CDS) and encodes 121 amino acids. The OsATG8c protein was localized to the cytoplasm. We then confirmed its function using *35S-OsATG8c* in transgenic rice plants. The independent transgenic lines were found to promote the effective tillering of rice and produce more ears and seeds, thus increasing the yield. Meanwhile, autophagic flux was significantly enhanced in the *OsATG8c*-overexpressors under both nitrogen sufficient and deficient conditions. It was also shown that the overexpression of *OsATG8c* significantly enhanced the nitrogen uptake efficiency (NUpE) and NUE under both N conditions. Therefore, our results indicate that *OsATG8c* may be an important candidate gene for rice with increased NUE and better grain yield potential.

## 2. Results

### 2.1. The Expression Patterns of the OsATG8c Gene in Rice

**Figure 1:**
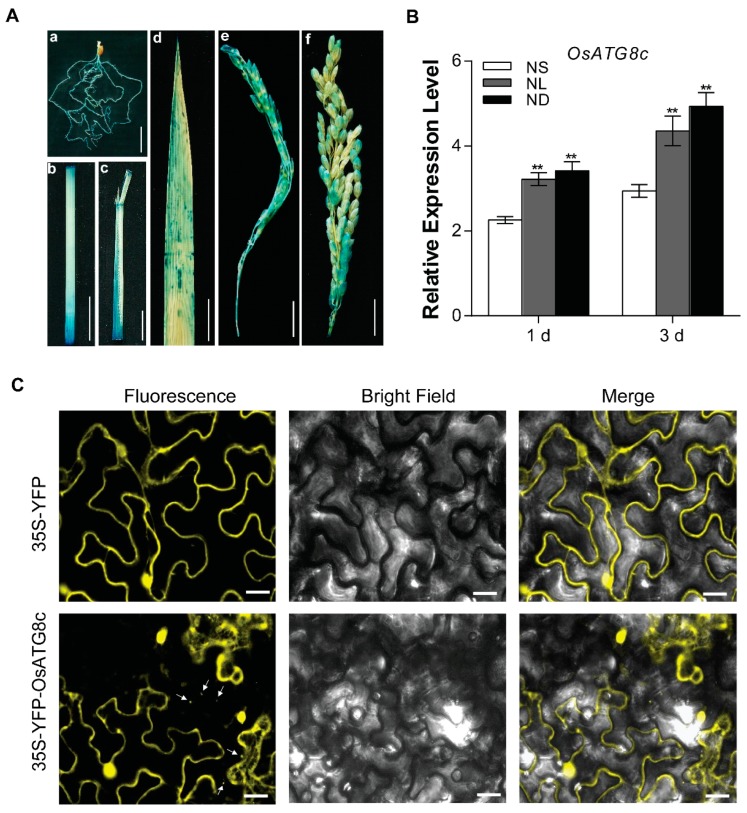
The expression patterns of the *OsATG8c* gene and protein subcellular localization of OsATG8c. (**A**) β-glucuronidase (GUS) histochemical staining of *Pro_OsATG8c_-GUS* transgenic rice. The tissues used for GUS staining included (a) root, (b) culm, (c) leaf sheath, (d) leaf blade, and panicle at the (e) booting stage and (f) grain filling stage. Scale bars: 2 cm in a–c and f, 1 cm in d and e. (**B**) The rice seedlings of Shennong9816 (SN9816) were cultured with sufficient nitrogen (N) (NS, 3.5 mM N) solution for 14 days and transferred to the same NS (3.5 mM N) solution as the control, low N (NL, 0.8 Mm N) solution and the deficient N (ND, 0 mM N) solution for 1 day and 3 days, respectively. When determining the expression of the *OsATG8c* gene in leaves, *OsActin1* was used as an internal control. Values are means ± SD (*n* = 10), ** *p* < 0.01 (*t*-test). (**C**) Subcellular localization of *OsATG8c* in tobacco leaves. From left to right, the panels show the images of yellow fluorescent protein (YFP) fluorescence and the bright-field and the YFP fluorescence and bright-field overlap image, respectively. 35S-YFP was used as a control. The white arrowheads indicate the autophagic structures. Scale bar: 10 μm.

Histochemical analysis was performed to examine the temporal and spatial expression patterns of *OsATG8c* in *Pro_OsATG8c_*-*GUS* transgenic rice. It was proved that β-glucuronidase (GUS) activity was detected in various rice organs, such as the roots of seedlings, culm, leaf sheaths, leaf blades, and young panicles at the booting stage, except that *OsATG8c* was also expressed in the panicles at the grain filling stage (Figure 1A). In order to analyze whether the expression of *OsATG8c* was responsive to N stress, the transcription level of *OsATG8c* was examined by real-time RT-PCR. The transcriptional level of *OsATG8c* in rice seedlings was significantly increased under both low N (NL) and N deficient (ND) conditions (Figure 1B). The fluorescence signals of YFP-OsATG8c were detected on the cytoplasm and autophagic structures in the tobacco (*Nicotiana benthamiana*) leaf cells (Figure 1C). 

### 2.2. Overexpression of OsATG8c Promotes Tillering in Transgenic Rice

**Figure 2:**
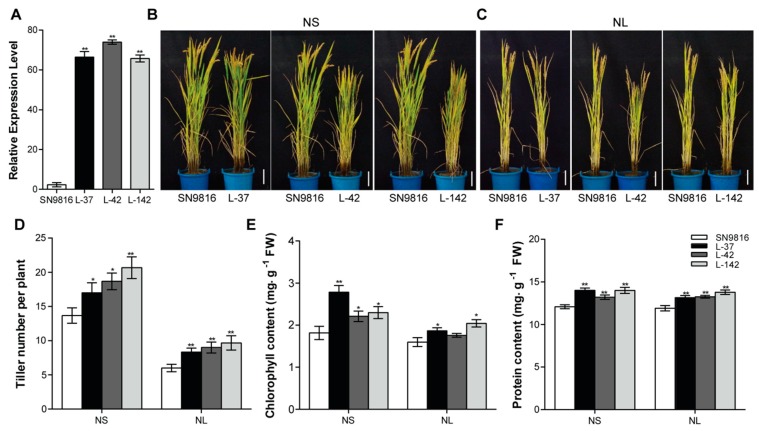
Characterization of *35S-OsATG8c* transgenic lines. (**A**) Expression level of *OsATG8c* in 14-day-old seedlings of SN9816 and transgenic lines (L-37, L-42 and L-142). *OsActin1* was used as an internal control. Values are the means ± SD (*n* = 3). (**B**,**C**) Phenotypes of the whole rice plants under sufficient N (NS, 225 kg·ha^−1^) or low N (NL, 75 kg·ha^−1^) conditions at the grain filling stage. Scale bar: 10 cm. (**D**) The tiller number per plant, (**E**) chlorophyll content, and (**F**) protein content of SN9816 and the transgenic lines under NS (225 kg·ha^−1^) and NL (75 kg·ha^−1^) conditions. Values are the means ± SD (*n* = 12), * *p* < 0.05, ** *p* < 0.01 (*t*-test).

**Figure 3:**
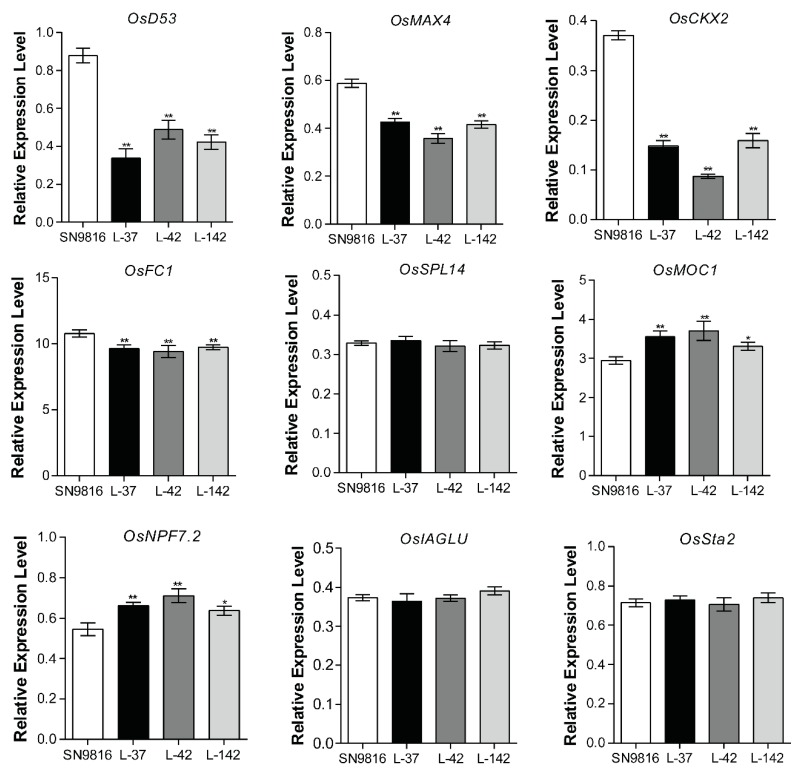
Overexpression of *OsATG8c* affected the expression of the tiller-related genes. The expression levels of strigolactone pathway genes (*OsD53* and *OsMAX4*), the cytokinin oxidase/dehydrogenase (CKX) enzyme coding gene *OsCKX2*, tiller inhibiting genes (*OsFC1* and *OsSPL14*), and tiller promoting genes (*OsMOC1*, *OsNPF7.2*, *OsIAGLU* and *OsSta2*) in the tiller buds of 32-day-old SN9816 and transgenic rice seedlings. Values are the means ± SD (*n* = 10), * *p* < 0.05, ** *p* < 0.01 (*t*-test).

The *35S*-*OsATG8c* fusion construct (Figure S1) was generated, and 17 independent *OsATG8c*-overexpressing transgenic lines were obtained. The transcription level of *OsATG8c* was confirmed in all transgenic lines using real-time RT-PCR, and 3 homozygous overexpressed lines (L-37, L-42, L-142) were randomly selected for further analysis (Figure 2A). The transcriptional levels of the other *OsATG*s were not changed in the *OsATG8c*-overexpressing transgenic rice (Figure S2). Under both normal growth (NS) and N-deficiency (NL) conditions, the transgenic lines showed increased tillering compared with that of the control plant Shennong9816 (SN9816) (Figure 2B–D). In addition, the chlorophyll content (Figure 2E) and protein content (Figure 2F) of the transgenic lines were more than that of the control at the grain filling stage. We further examined the expression levels of some tiller-related genes in rice tiller buds. It was found that the strigolactone pathway genes *OsMAX4* and *OsD53*, the cytokinin oxidase/dehydrogenase (CKX) enzyme coding gene *OsCKX2*, and the tiller inhibitor *OsFC1* were significantly down-regulated in transgenic lines compared with SN9816, whereas the tiller promoting genes *OsMOC1* and *OsNPF7.2* were significantly up-regulated (Figure 3). Moreover, another two strigolactone pathway genes (*OsD3* and *OsD27*) as well as three cytokinin degradation genes (*OsCKX1*, *OsCKX3*, and *OsCKX6*) were also down-regulated in transgenic lines (Figure S3). However, the expression level of the tiller inhibiting gene *OsSPL14* and two tiller promoting genes (*OsIAGLU* and *OsSta2*), as well as the strigolactone pathway genes (*OsD14* and *OsD17*) exhibited no significant differences among SN9816 and the transgenic lines (Figure 3 and Figure S3). In addition, the transgenic lines were shorter than those of the control plants at the jointing stage, and the plant height decreased by up to 15.28% or 10.53% compared with that of SN9816 under NS or NL conditions at the grain-filling stage (Figure S4A,B). The decreased plant height in transgenic lines was mainly caused by decreasing the length of the internodes (Figure S4C–E). 

### 2.3. Overexpression of OsATG8c in Transgenic Rice Enhances the Autophagic Flux

**Figure 4:**
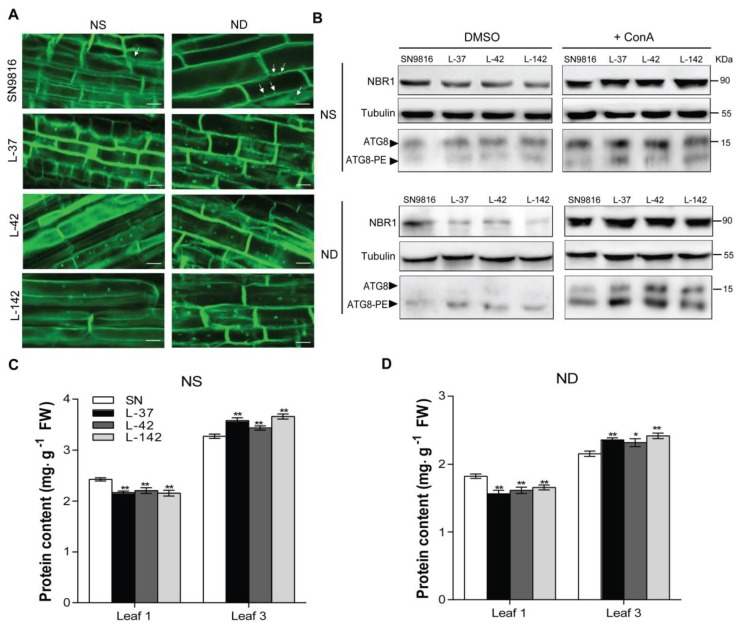
The overexpression of *OsATG8c* enhanced the autophagy levels and affected leaf protein profiles in rice. The 21-day-old seedlings of SN9816 and overexpressors were transferred to NS (3.5 mM N) or ND (0 mM N) liquid medium for 24 h. (**A**) Confocal analysis of autophagosomes in roots by monodansylcadaverine (MDC) staining. MDC-labeled structures are shown in green. The white arrowheads indicate the MDC-stained autophagosomes. Scale bars: 10 μm. (**B**) Fourteen-day-old seedlings were transferred to NS (3.5 mM N) and ND (0 mM N) liquid media with 0.5 µM concanamycin A (ConA) or solvent control DMSO for 24 h. Immunoblot analysis was used to determine the accumulation of NBR1 with anti-NBR1 in Arabidopsis; near-equal protein loads were confirmed with an α-tubulin antibody. The membrane fraction was used to detect lipidated (ATG8-PE) and free ATG8 levels with anti-Arabidopsis ATG8a in leaves. (**C**) The protein content of old leaves (Leaf 1) and (**D**) young leaves (Leaf 3) in SN9816 and transgenic rice under NS or ND conditions. The protein content in equal weight leaf samples was determined by bicinchoninic acid (BCA) protein assay, which was represented as mg protein/g fresh weight. Values are the means ± SD (*n* = 3), * *p* < 0.05, ** *p* < 0.01 (*t*-test).

To investigate whether the autophagic flux was enhanced in the *OsATG8c*-overexpressing transgenic rice, seedlings of the transgenic lines and control plants were treated with NS solution or ND solution for 24 h. We first examined the number of autophagosomes by monodansylcadaverine (MDC) staining. MDC is an acidophilic dye, which can be used to detect autophagosomes in plants and mammals [54,55,56]. It was observed that there were significantly more autophagosomes marked by MDC, while the intensity was also higher in the transgenic rice (Figure 4A). The Neighbor of BRCA1 (NBR1) protein is an autophagy receptor, and the substrate degrades in the vacuole of the cell. The degradation level of NBR1 is usually used to identify the selective autophagic flux in plants [57,58]. In addition, the degradation level of NBR1 protein was further investigated by Western blotting. The degradation of the NBR1 protein in transgenic lines increased, especially under N deficient conditions (Figure 4B, DMSO). Concanamycin A (ConA) was used to inhibit vacuolar acidification and autophagosome turnover. It was observed that ConA-mediated inhibition of vacuolar degradation greatly increased the NBR1 protein level (Figure 4B, +ConA). Previous reports showed that the Arabidopsis autophagy core protein, ATG8a, is an autophagic flux marker protein, as the lipidation of ATG8 can be used to indicate the autophagic activity [55,59,60]. The lipidated ATG8 (ATG8-PE) membrane protein was detected by Western blotting to analyze the autophagy flux. It was shown that the overexpression of *OsATG8c* significantly increased the accumulation of ATG8-PE in the membranes of transgenic lines; thus, the lipidation of ATG8 was enhanced, especially under N deficiency conditions and especially with ConA (Figure 4B). All of these results show that the autophagosome number was significantly greater in *OsATG8c*-overexpressing transgenic lines, and thus, the autophagic flux increased. This increased activity may be the reason for improving the tolerance to N deficiency stress. In plants, autophagy played an important role in maintaining protein profiles [15,16]. The protein content in old leaves (Leaf 1) and young leaves (Leaf 3) was compared under both NS and ND conditions. In leaf samples of the same quality, the protein content of old leaves in transgenic lines was lower than that in control plants under both two N conditions (Figure 4C), and identical leaves under the ND condition had much less protein (Figure 4D). Corresponding to that, the protein content of young leaves in transgenic lines was much higher than that of SN9816 (Figure 4C,D). These results show that the leaf protein content was affected by N stress and leafage. The autophagy level led to high remobilization and reuse of N and most proteins from old leaves into young leaves.

### 2.4. Overexpression of OsATG8c Increases the Yield and Promotes NUE in Transgenic Rice

**Figure 5:**
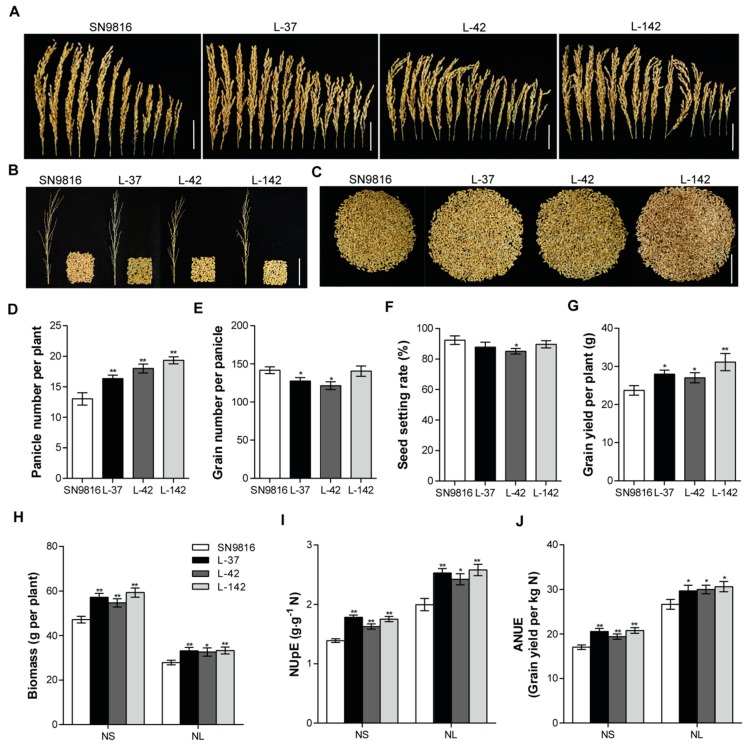
(**A**) The panicles per plant, (**B**) grains per panicle, (**C**) total grains per plant, (**D**) panicle number per plant, (**E**) grain number per panicle, (**F**) seed setting rate, and (**G**) grain yield per plant of SN9816 and *OsATG8c*-overexpressing transgenic rice grown under NS (225 kg·ha^−1^) conditions. Scale bar: 5 cm. Biomass per plant (dry weight of all aboveground) (**H**), nitrogen uptake efficiency (NUpE) (**I**), and nitrogen use efficiency (NUE) (**J**) of SN9816 and *OsATG8c*-overexpressing lines grown under NS (225 kg·ha^−1^) and NL (75 kg·ha^−1^) conditions. Values are the means ± SD (*n* = 12), * *p* < 0.05, ** *p* < 0.01 (*t*-test).

We further investigated the effect of *OsATG8c* on the yield and found that the transgenic lines produced significantly more panicles per plant (Figure 5A), and there was little difference in the panicle type compared with that of the control plants under the full N condition (Figure 5B). However, the grain number per panicle seed setting rate was reduced (Figure 5E,F), while the panicle number per plant significantly increased (Figure 5D). As a result, the transgenic lines had a significantly higher total grain yield per plant than the controls (Figure 5C,G). Corresponding to the higher yield in transgenic lines, the biomass also increased up to 25.72% and 19.49% under NS and NL conditions, respectively (Figure 5H). Since the biomass significantly increased in the *OsATG8c*-overexpressing transgenic lines, both the NUpE and NUE were higher under both NS and NL conditions (Figure 5I,J), suggesting that *OsATG8c* might be essential for both grain yield and NUE. 

## 3. Discussion

Autophagy takes part in the nutritional cycle of all eukaryotes and also acts as a pivotal part in the remobilization and transfer of N from old leaves to grains in plants [61]. Previous studies have also shown that many *ATG* genes are transcriptionally up-regulated under nutrient-deficient conditions, and enhanced autophagy also occurs [46,47,62,63,64]. In this study, the core gene of autophagy in rice, *OsATG8c*, was preferentially expressed after three days of N starvation (both ammonium and nitrate starvation or even free) (Figure 1B), indicating that *OsATG8c* may also confer tolerance to this stress in rice. However, earlier studies also investigated the expression pattern of *OsATG8c* subjected to ammonium or nitrate starvation and, except for a slight reduction after 24 h of ammonium starvation, there were no obvious expression changes [46]. The differences shown in our results might be due to the different experimental processing conditions and degree of nitrogen starvation used. Xia et al. performed semi-quantitative RT-PCRs to detect the *OsATG8c* expression profile in rice at the booting and the seedling stage [46]. Furthermore, we reported the temporal and spatial profiles of the expression of *OsATG8c* by histochemical staining (Figure 1A), and the result was supplementary to the previous results.

Here, we further demonstrated the effect of *OsATG8c* on rice growth and yield. It was found that the overexpressed *OsATG8c* could not only promote the growth of rice but also restore growth inhibition due to nitrogen deficiency in the transgenic rice (Figure 2C). As the transcriptional levels of other *OsATG*s were not affected in the *OsATG8c*-overexpressing rice (Figure S2), we considered that the phenotypes of the transgenic lines were determined by *OsATG8c*-mediated increased autophagy. The *35S-OsATG8c* transgenic lines not only grew better than SN9816 under N-limited conditions, but they also performed better under normal growth conditions. The transgenic rice plants overexpressing *OsATG8c* had increased chlorophyll and protein contents in the leaves (Figure 2E,F), as well as greater biomass accumulation (Figure 5H). During the vegetative growth stage, the transgenic lines showed greater accumulation of the photosynthetic products, and this would be more N for plants to use in reproductive growth and yield.

Interestingly, after statistical analysis, it was found that the plant height of the *OsATG8c*-overexpressing rice decreased (Figure S4). In agronomy, plant height becomes shorter, which is a good characteristic for production and is considered the ideal plant type. Furthermore, the overexpression of *OsATG8c* significantly promoted effective rice tillering and increased yield (Figure 5A,C). Although the grain number per panicle reduced in the *OsATG8c*-overexpressing lines (Figure 5E), the panicle number per plant was more than that of the control, so the yield still increased (Figure 5D,G). It has been reported that *OsMOC1* and *OsNPF7.2* promote rice tillering [25,38], while *OsFC1* inhibits tillering [26]. In the *OsATG8c*-overexpressing transgenic rice, the expression levels of *OsMOC1* and *OsNPF7.2* were increased, but the expression of *OsFC1* was decreased (Figure 3), which finally resulted in an increased tillers number. Besides, the biosynthesis and signaling related genes of strigolactones and cytokinin oxidase/dehydrogenase (CKX) enzyme coding gene *OsCKX2* were reported to be involved in the tillering regulation in rice [32,33,35]. In this study, the overexpression of *OsATG8c* down-regulated the strigolactone synthesis genes (*OsMAX4*, *OsD27*) and signaling genes (*OsD3*, *OsD53*), as well as the cytokinin degradation genes *OsCKX*s in transgenic lines (Figure 3 and Figure S3), which suggests that the cytokinin and strigolactone pathways might be involved in the function of *OsATG8c* in promoting rice tillering. As rice tillering is an important determinant of the panicle number and grain yield [22], the increased tiller and panicle number in the *35S-OsATG8c* transgenic rice resulted in an increase in yield. Meanwhile, GUS histochemical staining showed that *OsATG8c* was expressed in the developing panicles (Figure 1A), suggesting that the *OsATG8c* gene may also be involved in grain formation in rice. 

Studies on some *atg* deletion mutants of Arabidopsis, maize, and rice have shown diminished growth and development, accelerated senescence, a decreased number of autophagosomes, and decreased autophagy activity, as well as decreased N remobilization from leaves to seeds [9,11,13,15,65]. As shown in Figure 4A,B, the increased autophagosomes in transgenic rice and the enhanced ATG8a lipidation in *OsATG8c*-overexpressing lines, particularly under N deficiency condition, as well as the accumulation of NBR1 with/without ConA, all suggest that autophagic flux was significantly stimulated in transgenic rice. In addition, since the degradation of protein occurred in response to leaf senescence, it was also detected that the degradation of proteins during leaf senescence was enhanced in the transgenic rice, confirming that the autophagy level was raised in another aspect (Figure 4C,D). As a result, the autophagy activity was significantly promoted in *OsATG8c*-overexpressing transgenic rice, thereby increasing the tolerance to N stress. On the other hand, the advanced autophagy activity phenotype extended the remobilization of nutrients, resulting in an increased number of panicles and, consequently, a better yield (Figure 5A,C,G). Autophagy is a facilitator of nutrient remobilization in plants, and it also plays a vital role in improving the NUE and crop yield protection [66]. The redundant N in the cells cannot be effectively recovered or utilized, leading to nutrient accumulation and wastage, cell death, and a lower amino acid content, all of which suppress the growth and reduce the NUE of plants with defective autophagy [67]. Our contemporaneous research showed that the overexpressed *OsATG8a* in transgenic rice not only increased the autophagic flux, but also had a significant phenotypic change on vegetative growth and fecundity [53]. Although *OsATG8a* and *OsATG8c* belong to a subfamily in rice, their phylogenetic distance was large, indicating that the divergence of these two genes might have occurred very early, so we speculated that their functions may be different. Overexpression of *OsATG8a* significantly increased NUE and NUpE under normal N conditions, but did not seem to have any effect on NUE under suboptimal N conditions. Interestingly, it was shown that the NUpE and NUE of transgenic rice were higher under the two N conditions. In addition, overexpression of *OsATG8a* in rice might promote the remobilization of proteins from leaves into seeds. While, overexpression of *OsATG8c* could promote the remobilization and reuse of proteins from old leaves to young leaves. The increased grain yield was at least partially caused by effective remobilization of assimilated N from the vegetative tissue to the developing seeds, presumably due to enhanced N uptake and N recycling by autophagy. Although the NUpE and NUE of transgenic rice were both increased under the two N conditions, we did not observe an increased yield under the N deficiency condition. A possible reason for this might be due the harsh N deficiency condition. The low nitrogen condition (NL) that we used in this research was a two-thirds N deficiency condition containing only a one-third of the N supply compared to that of the NS treatment, which was already a very harsh condition. The limited N might have contributed to the maintenance of normal plant growth and enhanced the resistance to N stress. Since autophagic recycling is required for nutrient mobilization, the yield increment observed in the transgenic lines could be a benefit of the enhanced autophagic recycling ability, leading to efficient N cycling, as nutrients are remobilized from the leaves to the reproductive organs (grains). Even under a very harsh N deficiency (NL) condition, compared with the control rice, transgenic rice still had enhanced NUpE and NUE. This indicates that the *OsATG8c* gene has great potential to be of benefit in agricultural production.

## 4. Materials and Methods 

### 4.1. Plant Materials and Growth Conditions

The *japonica* (*Oryza sativa*) cultivar Shennong9816 (SN9816), the main variety of Shenyang in northeast China, was used as control plant. Rice seedlings were grown hydroponically in a growth chamber (28 °C/25 °C and 10 h light/14 h dark), with modified half Hoagland’s solution containing different N concentrations (pH = 5.7) as previously described [51] for short-term treatment. 

### 4.2. Binary Vector Construction

To produce the *35S-OsATG8c* and *35S-YFP-OsATG8c* fusion constructs, the *OsATG8c* complete coding region was amplified and cloned into the binary vectors pCAMBIA1301 and pCAMBIA1300-YFP. A 1405-bp promoter fragment of the *OsATG8c* was amplified and cloned into pCAMBIA1301 vector to construct the *Pro_OsATG8c_-GUS* fusion gene. The corresponding primers are listed in Table S1 and all the fusion constructs were confirmed by sequencing. 

### 4.3. Gene Expression Analysis

To analyze *OsATG8c* expression responsive to N stress in rice, rice seedlings were grown for 14 days in half Hoagland’s solution (NS, 3.5 mM N), then supplied with the same NS solution as the control, low N (NL, 0.8 mM N) solution (with 0.6 mM KNO_3_ and 0.1 mM (NH_4_)_2_SO_4_, pH = 5.7) and the N-free (ND, 0 mM N) solution, KCl was used to supplement the lack of K^+^. The 21-day-old seedlings of SN9816 and *35S-OsATG8c* transgenic lines cultured with NS solution were sampled for expression analysis of the other *OsATGs.* To analyze the expression level of tiller related genes, tiller buds were obtained from the 32-day-old rice seedlings of SN9816 and *35S-OsATG8c* transgenic lines. Total RNA extraction, cDNA synthesized and real-time RT-PCR were conducted as previously described [53]. *OsActin1* was used as an internal control. The corresponding primers used for gene expression analysis are listed in Table S1. All experiments were subjected to 3 biological replicates.

### 4.4. Protein Isolation and Immunoblot Analysis, Rice Transformation, MDC Staining, Measurement of Chlorophyll, Soluble Protein Content, and Analysis of Agronomic Traits, Biomass, NUpE and NUE Calculations

All of these were performed as described previously [53]. 

### 4.5. Field Trials of Rice

*35S-OsATG8c* transgenic rice (T_3_ generation) and SN9816 were grown under natural growth condition at Shenyang Agricultural University experimental farm (Shenyang, China; Longitude: 123.34°E Latitude: 41.49°N). Field trials for N fertilizer treatments were conducted as described previously [53]. 

### 4.6. GUS Histochemical Staining

*Pro_OsATG8c_-GUS* transgenic rice was used to analyze the temporal and spatial expression patterns. GUS histochemical staining was performed as described in [68], in tissues from the root, culm, leaf sheath, leaf blade, and panicle of *Pro_OsATG8c_-GUS* transgenic rice. 

### 4.7. Subcellular Localization Assay

The subcellular localization analysis of OsATG8c was conducted with the *35S-YFP-OsATG8c* fusion constructs. The sequence-verified construction was introduced into the *Agrobacterium* strain GV3101 and then used to infiltrate tobacco leaves according to published methods [69]. The YFP fluorescence was observed by confocal microscopy (Zeiss LSM 780, Carl Zeiss, Jena, Germany). 

### 4.8. Statistical Analysis 

Data comparisons between transgenic lines and SN9816 were statistically analyzed by Student’s *t*-test at significance levels of * *p* < 0.05 and ** *p* < 0.01. 

## Supplementary Materials

The following are available online at https://www.mdpi.com/1422-0067/20/19/4956/s1.

## Abbreviations

ATG	Autophagy
NUE	Nitrogen use efficiency
NUpE	Nitrogen uptake efficiency
MDC	Monodansylcadaverine
ConA	Concanamycin A
