# Supplementary material for: OsATG8c-Mediated Increased Autophagy Regulates the Yield and Nitrogen Use Efficiency in Rice
Source: Int J Mol Sci. 2019 Oct 8;20(19):4956. doi: 10.3390/ijms20194956 (PMC6801700; doi:10.3390/ijms20194956)
Supplement: Supplementary file 1 [file ijms-20-04956-s001.pdf]

Supplementary Materials

OsATG8c-mediated increased autophagy increases the yield and nitrogen use efficiency (NUE) in rice

Xiaoxi Zhen[1] · Xin Li[1] · Jinlei Yu[1] · Fan Xu[1,*]

1 Rice Research Institute of Shenyang Agricultural University, Key Laboratory of Northern Japonica Rice Genetics and Breeding, Ministry of Education and Liaoning Province, Key Laboratory of Northeast Rice Biology and Genetics and Breeding, Ministry of Agriculture, Shenyang 110866, China; xiaoxizhen1991@163.com (X.Z.); lixin241910@163.com (X.L.); yujinlei0628@163.com (J.Y.)

*Correspondence: celiafanfan@163.com or celiafanfan@syau.edu.cn (F.X.)

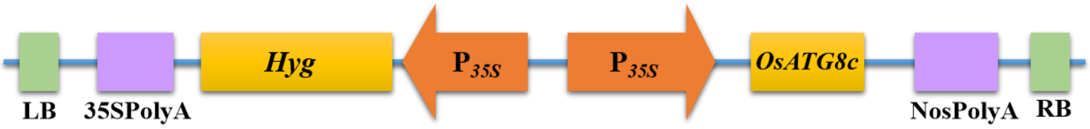

Supplementary Figure 1. Schematic diagram of *35S-OsATG8c* construction. P_{35S}, CaMV 35S promoter; *Hyg*, hygromycin; 35SPolyA; CaMV 35S terminator polyA signal; NosPolyA, nopaline synthase terminator polyA signal; RB and LB, right and left border of Ti plasmid.

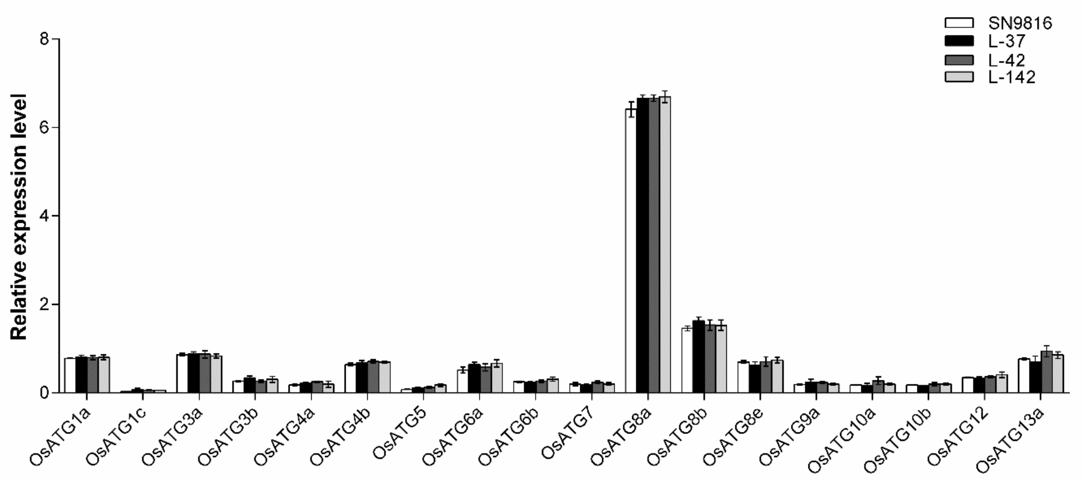

Supplementary Figure 2. Real-time RT-PCR analysis of the transcript levels of *OsATGs* in 21-day-old seedlings of SN9816 and *35S-OsATG8c* transgenic rice under N sufficient condition.

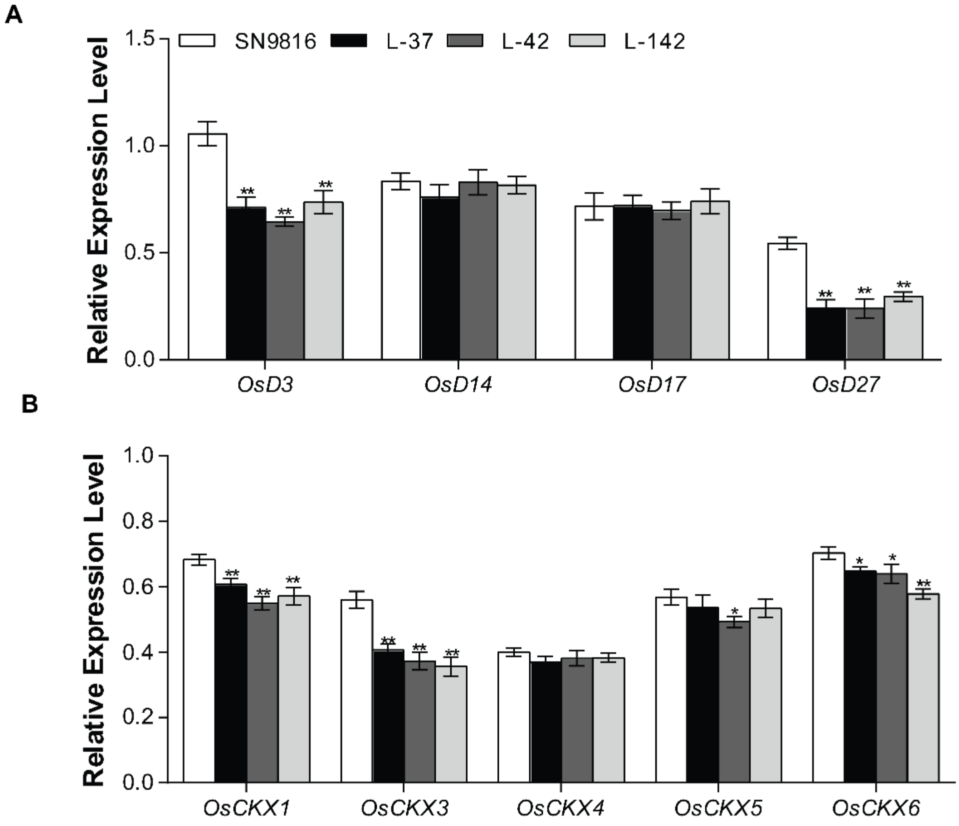

Supplementary Figure 3. Overexpression of *OsATG8c* affected the expression of the tiller related genes. Expression level of (A) genes involved strigolactones pathway and (B) cytokinin

oxidase/dehydrogenase (CKXs) genes in tiller buds of 32-day-old SN9816 and transgenic rice seedlings. Values are means ± SD (n = 10), **P* < 0.05, ***P* < 0.01 (t-test).

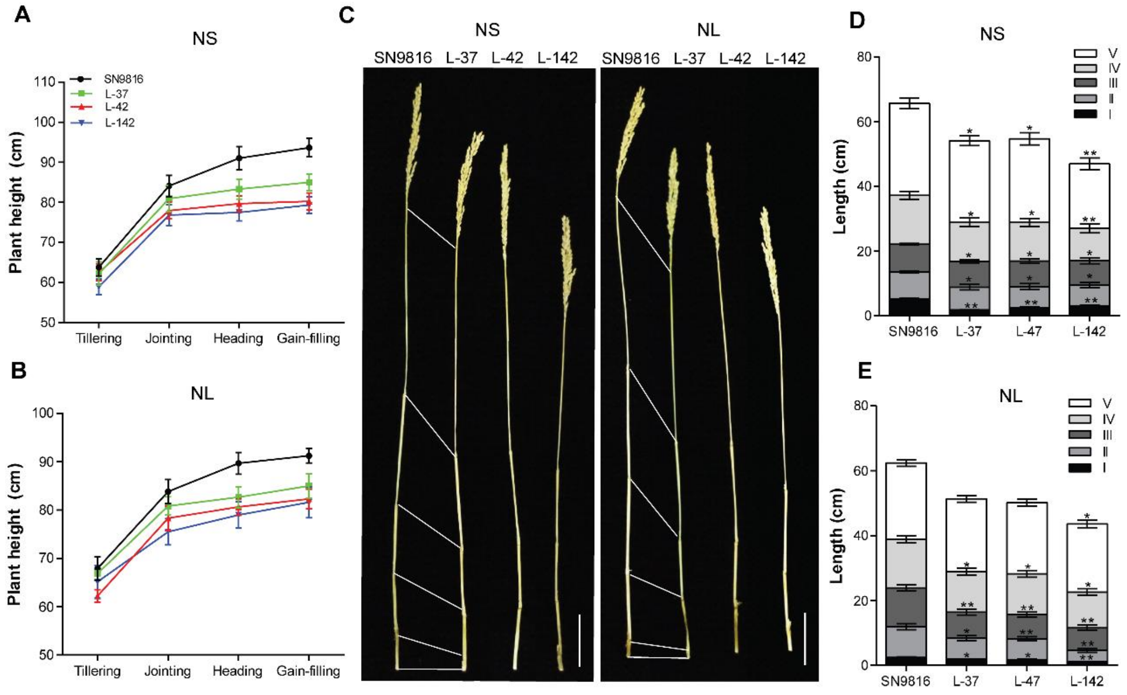

Supplementary Figure 4. Overexpression of *OsATG8c* lowered plant height. (A) and (B) The plant height curve of SN9816 and transgenic lines under NS (225 kg·ha^{-1}) and NL (75 kg·ha^{-1}) conditions at various growth stage, values are means ± SD (n = 12). (C) Comparisons of culm length in SN9816 and transgenic lines under NS (225 kg·ha^{-1}) and NL (75 kg·ha^{-1}) conditions at ripening stage. I, II, III, IV and V indicate the first, second, third, fourth and fifth internode, respectively. Scale bars, 10 cm. (D) and (E) Quantification of the internode length in panel C, values are means ± SD (n = 12), asterisks indicate significant differences of each internode length between SN9816 and the transgenic lines, **P* < 0.05, ***P* < 0.01 (t-test).

Supplementary Table 1 The information of primers used in this study.

Primer name	Sequence (5′→ 3′)	Functions
cOsATG8c-F	CCATGGATGGCGAGGAGCTCGTTCAAGC	Gene cloning
cOsATG8c-R	GGTGACCTCAGACAAACAGGCCGAAGGTGT	Gene cloning
cpOsATG8c-F	AAGCTTCGTGGCTGTGTATGCCTTGC	Gene cloning
cpOsATG8c-R	CCATGGCGCCCTGCCTTGCTTTCTT	Gene cloning
rtOsATG8c-F	GACCTCACGGTTGGACAGTT	Real-time RT-PCR
rtOsATG8c-R	TGGAAGCGTGTTCTTGACGA	Real-time RT-PCR
rtOsATG1a-F	CCAAACGAGGGCGTGTAAGA	Real-time RT-PCR
rtOsATG1a-R	CACCGAGCTCACGTTACAGT	Real-time RT-PCR
rtOsATG1c-F	TGCTCCTGCACACGGTTATT	Real-time RT-PCR
rtOsATG1c-R	TCCATAGGGGGACATGAGCA	Real-time RT-PCR
rtOsATG3a-F	AGCCAAGATCATGCACGGAA	Real-time RT-PCR

rtOsATG3a-R	ACAGCAGCATGTTTGCATGG	Real-time RT-PCR
rtOsATG3b-F	CTGCCCCTCTCTTGCACAAT	Real-time RT-PCR
rtOsATG3b-R	CCAAATACAGGCATGCGACC	Real-time RT-PCR
rtOsATG4a-F	GCCGTGACAAAGGTGAACTG	Real-time RT-PCR
rtOsATG4a-R	CTCAGAGGCCCGAGAACAGA	Real-time RT-PCR
rtOsATG4b-F	GCACTGTCTCGATACCCCAG	Real-time RT-PCR
rtOsATG4b-R	CGCTGTAACATGGTAACAGAACG	Real-time RT-PCR
rtOsATG5-F	CAGCTCGAAGGCATCACGTA	Real-time RT-PCR
rtOsATG5-R	TGCCTTGCACCCTTACTAGC	Real-time RT-PCR
rtOsATG6a-F	GTGACAAGGTAGGGAGCCAC	Real-time RT-PCR
rtOsATG6a-R	GATTGCGCTGCACACAAAGA	Real-time RT-PCR
rtOsATG6b-F	TGCAGCTCTGGTTAGGTGAG	Real-time RT-PCR
rtOsATG6b-R	TCTCAAGTATGTGTTGCTGAAGAC	Real-time RT-PCR
rtOsATG7-F	AGCCTGCGTTGGAAGATTGA	Real-time RT-PCR
rtOsATG7-R	GGAAATGATGCTTCGCCGAC	Real-time RT-PCR
rtOsATG8a-F	ATAGCATGTCCCTGTGCTGG	Real-time RT-PCR
rtOsATG8a-R	CGCCCAATCACACTTCAGAC	Real-time RT-PCR
rtOsATG8b-F	GCTGATCTTACCGTTGGGCA	Real-time RT-PCR
rtOsATG8b-R	ATCAGAGCAGCTGTTGGTGG	Real-time RT-PCR
rtOsATG8e-F	CCTCCGTGGAGATCTGCTTT	Real-time RT-PCR
rtOsATG8e-R	GGTGGGATAATTAAGTTCGGTATG	Real-time RT-PCR
rtOsATG9a-F	ATGGCGATGAAACGTCGGAT	Real-time RT-PCR
rtOsATG9a-R	CTGTCCTTCTGAGGGGCATC	Real-time RT-PCR
rtOsATG10a-F	GTGAGGGATGGGACTCTGTC	Real-time RT-PCR
rtOsATG10a-R	TGGAACTCCCATCTTGCAGC	Real-time RT-PCR
rtOsATG10b-F	TTCCAGGCTCCACTGCTCT	Real-time RT-PCR
rtOsATG10b-R	CAAACACAGGATTTGATGGCTC	Real-time RT-PCR
rtOsATG12-F	AGTCCAAGTTCAAGATTGGTGGA	Real-time RT-PCR
rtOsATG12-R	GTTGCCCGTCGATTCCAAAG	Real-time RT-PCR
rtOsATG13a-F	GTCAACCGGCGCTTGTTATG	Real-time RT-PCR
rtOsATG13a-R	ATCCGGTTGGTTTTGCTTGC	Real-time RT-PCR
rtOsD3-F	TCCAAACTTGCGGGACATGCAGTT	Real-time RT-PCR
rtOsD3-R	CCATTGCACAGTGGAGCAATGGCA	Real-time RT-PCR
rtOsD10-F	CTGTACAAGTTCGAGTGGCACC	Real-time RT-PCR
rtOsD10-R	CCTCGTCCGTCTCCTCGTAC	Real-time RT-PCR
rtOsD14-F	GTGCTGTCGCATGGCTTC	Real-time RT-PCR
rtOsD14-R	GCAGGTCGTCGACGTAGG	Real-time RT-PCR

rtOsD17-F	GAGGATGGTGGCTATGTTCT	Real-time RT-PCR
rtOsD17-R	AGACTGGATCTGATGCTTGCT	Real-time RT-PCR
rtOsD27-F	TCTGGGCTAAAGAATGAAAAGGA	Real-time RT-PCR
rtOsD27-R	AGAGCTTGGGTCACAATCTCG	Real-time RT-PCR
rtOsD53-F	TGGGTTCTGAGTGCATGTTG	Real-time RT-PCR
rtOsD53-R	GTGTCCTCACAGGCCACTAG	Real-time RT-PCR
rtOsCKX1-F	TCAACAAATCCAAGTGGGATGCGG	Real-time RT-PCR
rtOsCKX1-R	TCGCAGAACCTCAGTATCCTCCTGT	Real-time RT-PCR
rtOsCKX2-F	GTCAGTGGAGGGGCGGTA	Real-time RT-PCR
rtOsCKX2-R	CGTTGGAAATCTGGGGGC	Real-time RT-PCR
rtOsCKX3-F	ATGAGCAATCCCTTCACAGCTCCT	Real-time RT-PCR
rtOsCKX3-R	TGACTTCCACGACCTGTTCCACAT	Real-time RT-PCR
rtOsCKX4-F	GACCGACTACCTCCATCTCACA	Real-time RT-PCR
rtOsCKX4-R	GGTTGACATTGCTGACCTGC	Real-time RT-PCR
rtOsCKX5-F	AGGGCCTAATCAACAACTGGAGGT	Real-time RT-PCR
rtOsCKX5-R	GGTGGAGTCGTCGTAATTCTTGGT	Real-time RT-PCR
rtOsCKX6-F	ACAAGTCCAGCTCAATCGGACACT	Real-time RT-PCR
rtOsCKX6-R	TCCCGCAAGCCTATCAATGTCGAA	Real-time RT-PCR
rtOsSPL14-F	GATGGATTGGTCTCTGTAGAGG	Real-time RT-PCR
rtOsSPL14-R	TTGAACACAAAATAAGGGCAGG	Real-time RT-PCR
rtOsFC1-F	GCTTGGAGCAGTGTACTATACT	Real-time RT-PCR
rtOsFC1-R	CTATGTCGATGCACACATTCAG	Real-time RT-PCR
rtOsMOC1-F	GTACCTGGCGTTCAACCA	Real-time RT-PCR
rtOsMOC1-R	TCGAGGTCGAGGATGTGGA	Real-time RT-PCR
rtOsSta2-F	GCTTGGAGCAGTGTACTATACT	Real-time RT-PCR
rtOsSta2-R	CTATGTCGATGCACACATTCAG	Real-time RT-PCR
rtOsNPF7.2-F	TGCAAGTGCCACTCCTCAAGG	Real-time RT-PCR
rtOsNPF7.2-R	AGGACGGTCTCCAGGTACACCACC	Real-time RT-PCR
rtOsIAGLU-F	TGCTCTTCAACTCGTTCGAC	Real-time RT-PCR
rtOsIAGLU-R	GACAACCCAGCCAAAACATTAA	Real-time RT-PCR
rtOsActin1-F	ACCATTGGTGCTGAGCGTTT	Real-time RT-PCR
rtOsActin1-R	CGCAGCTTCCATTCCTATGAA	Real-time RT-PCR
